# Development of an Emergency Clinical Handover Tool for Doctors Using the Fuzzy Delphi Method

**DOI:** 10.21315/mjms-09-2025-615

**Published:** 2026-04-30

**Authors:** Ismail Mohd Saiboon, Ahmad Khalil Ibrahim, Abdul Karim Mustafa, Mohd Johar Jaafar, Mohd Hisham Isa

**Affiliations:** Emergency Department, Faculty of Medicine, Universiti Kebangsaan Malaysia, Cheras, Kuala Lumpur, Malaysia

**Keywords:** patient handoff, emergency service, hospital, Delphi technique, patient safety

## Abstract

**Background:**

Clinical handover in emergency departments (EDs) is a critical, high-risk process, and miscommunication can lead to serious patient harm. Although the Introduction, Situation, Background, Assessment, Recommendation (ISBAR) framework is widely adopted to guide handovers, Malaysia currently lacks a validated and standardised tool specifically designed for ED settings. This gap underscores the need for a structured approach to ensure safer, more consistent patient transitions.

**Methods:**

To develop a new emergency clinical handover (ECH) tool, the Fuzzy Delphi Method (FDM) was employed with input from a panel of emergency physicians (EPs). A deductive approach was used to extract potential handover items, which were then rated on a 7-point Likert scale. These ratings were converted into triangular fuzzy numbers. Items were retained if they met three criteria: i) threshold value (*d*) ≤ 0.2; ii) expert agreement ≥ 75%; and iii) fuzzy score (*A*) ≥ 0.5. Perceptions of the proposed ECH tool were evaluated using a 10-item, 3-point Likert scale by the ED doctors after a one-month trial in a real-world ED situation.

**Results:**

The expert panel agreed on 19 items for inclusion in the ECH tool. These items were distributed across the ISBAR domains: five in Identification/Information, two in Situation, two in Background, seven in Assessment/Action, and three in Recommendation. The perception score indicated an acceptance rate of 70.17% among participants.

**Conclusion:**

The FDM effectively facilitated consensus in developing a structured ECH tool. This standardised approach may enhance communication, reduce variability, and improve patient safety during ED handovers.

## Introduction

Patient care in the hospital setting is a 24-hour-a-day requirement. However, the medical staff managing the patient will change periodically. Thus, the patient’s care needs to be handed over or handed off from one caregiver to another, regardless of the working system, whether the “on-call system” or the “shift system.” Both systems use a handover routine to ensure patient care remains at an optimal level. The shift system entails more handovers than the on-call system, with three to five per day (1). Worldwide, most, if not all, emergency departments (EDs) use a shift system. In essence, a good handover is crucial to maintain optimal patient care and ensure that vital information is relayed effectively at every handover.

A poor handover will result in issues such as delayed diagnosis, unnecessary repetition of investigations, gaps in management, instability once transferred to the wards, and patient dissatisfaction (2). The busier the shifts, the more frequent issues arise in the handover communication (3). Therefore, adaptation to a standardised system, such as the Situation, Background, Assessment/Action, Recommendation/Response (SBAR), a method for handover introduced in 2002, has been implemented. It has also been well received by nursing staff worldwide (4). Moreover, the SBAR method has been adapted by emergency physicians (EPs) into various handover tools. Over the years, this method evolved into ISBAR, which added the domain of Identification/Information and was recommended in 2009 (5). Despite the ISBAR method serving as a basis for handover, no standardised tool has been developed to date (6). Thus, it is widely proposed that, to prevent discontinuity in the patient’s care during handover, a standardised guideline should be established for physicians (2).

To avoid handover errors, a standardised handover protocol is needed. To the best of the authors’ knowledge, published studies on a standardised handover tool in Malaysian EDs are scarce. Therefore, the present study set out to evaluate and develop a handover tool that is user-friendly and easy to use in the ED setting, based on the ISBAR domains, using the Fuzzy Delphi Method (FDM), a structured, iterative process for gathering and synthesising expert opinions. Notably, FDM is a combination of the fuzzy number set and the Delphi method, in which the respondents must be experts in a field appropriate to the study’s context (7). Specifically, this study aims to: i) identify and determine the critical items required in developing this emergency clinical handover (ECH) tool, using FDM and ii) evaluate the perception and acceptance among ED medical officers of the newly developed ECH tool. This study hopes that a more robust tool can be developed for ED clinical handover.

## Methods

### Study Design

This is a prospective mixed-methods study conducted at a tertiary teaching hospital in the Klang Valley between July 2023 and November 2024. Accordingly, the study was divided into two phases. In phase one, a qualitative approach was used to create the ECH tool via FDM. Meanwhile, in phase two, a quantitative method was used to evaluate the perceptions of the new ECH tool.

### The Study Protocol

This study has two phases, in which phase one identifies the expert panel. The panel then identified the critical items from the existing list, developed through a deductive process. The items for the deductive method were not derived solely from a single ISBAR template. Nevertheless, they were synthesised from a comprehensive literature review of various handover guidelines and ISBAR adaptations used globally. While the ISBAR framework served as the structural domain, the specific items populated within these domains were identified through the deductive process. This preliminary list of 20 items was presented to the expert panel during the first round of interviews. Correspondingly, they could accept, reject, or suggest new items to ensure the content was comprehensive and relevant to the local ED context. The panel then scores the items using a Likert scale.

The expert engagement was conducted using a hybrid approach, consisting of an initial phase, during which the experts were interviewed via face-to-face sessions to discuss and refine the preliminary items. Subsequently, during the evaluation phase, the 7-point Likert scale questionnaire was distributed via Google Forms, with links disseminated via email and WhatsApp to ensure accessibility and convenience for the experts. The experts responded to the questionnaire within two weeks. Subsequently, the data were transformed using Fuzzy Delphi Numbering. Concurrently, the FDM analysis was performed on the data. The FDM results were used to form the ECH tool.

Phase two begins by selecting and enrolling the participant for the perception study. This was to assess the newly developed ECH tool in a real-world ED scenario. A total of 30 medical officers were enrolled using convenience sampling. Although a formal power calculation for a randomised trial was not applicable, the sample size was determined using established guidelines for face validity studies (8). This indicates that this sample size provides sufficient power to establish consensus and usability benchmarks for a new clinical tool. Consents were obtained from the qualified participants through convenient sampling. Participants were briefed on the protocol and used the ECH tool for one month during their clinical shift. At the end of the month, participants were asked to complete the perception questionnaire. The present study developed a 10-item perception questionnaire specifically to assess the face validity (clarity, feasibility, and relevance) of the ECH tool. To ensure the instrument’s reliability, a pilot study was conducted with 10 medical officers prior to the main study. Moreover, data from this pilot group were used to calculate the population mean (μ) and standard deviation (SD, σ), establishing a baseline Z-score benchmark for validating the tool’s acceptance in the subsequent full-scale study. The study flow is displayed in Figure 1.

### Phase One

In phase one, the sample population consisted of EPs who served on the expert panel. The inclusion criteria for the expert panel were EPs who have been working in the ED for more than two years and who are actively involved in patient handover. Conversely, EPs who are not actively involved in the clinical handover or those who are doing attachments are excluded from this study.

The sample size for the expert panel in this study was fixed at 10, based on Adler and Ziglo (9), who suggested that Delphi panels with 10 to 12 subject-matter experts are sufficient. They were recruited using convenience sampling.

#### Fuzzy Delphi Method

The FDM comprises nine steps:

- determining the number of experts required to form the expert panel;
- determine the range of the Likert scale and assign the rubric;
- develop the items of the ECH tool through a deductive process;
- evaluation of the item in the ECH tool by the expert panel according to the Likert scale;
- conversion score of the Likert scale into fuzzy numbers;
- calculation of the *d*-value of each item from the fuzzy numbers;
- calculating the percentage of each item based on expert consensus;
- calculating the Fuzzy *A* value of each item; and
- finalising the accepted and rejected items based on all three factors (*d*-value, percentage of consensus, and Fuzzy *A* value) obtained from steps (vi), (vii), and (viii).

Determining the range of the Likert scale, and rubric, also known as linguistic criteria (step [ii]), a 7-point Likert scale was chosen, according to Mohd Jamil and Mat Noh (10), as it provides a higher level of consensus of 90%. In step (v), the conversion of the Likert scale into fuzzy numbers was achieved through the triangular fuzzy number (refer to Figure 2a). For step (vi), the first requirement to be followed is that the threshold *d*-value must be less than or equal to 0.2 (10). Calculation of the *d*-value is according to the formula in Figure 2b. In step (vii), the percentage of expert agreement must be 75.0% or greater (11, 12). In this process, the second condition is determined by calculating the percentage of expert agreement. The Fuzzy *A* value (step [viii]) must be greater than or equal to the median value (α − cut value) of 0.5 (13). The formula below is used to calculate the value of Fuzzy *A*:


$$A=(1/3)*(m_{1}+m_{2}+m_{3}),$$

where *m* are fuzzy numbers, and cross-reference the result to Figure 2c.

Finally, the ninth step involves presenting the results of data analysis in a table, with displayed values and cells appearing in red indicating non-compliance with the conditions set by the FDM. This suggests that expert agreement rejects those elements, providing a clear summary of the expert panel’s consensus.

### Phase Two

In phase two, a quantitative study of the end user (ED doctors) perception of the ECH tool was conducted. The inclusion criteria were all medical officers working in the department during the study period who had worked in the ED for more than two years. Those ED medical officers who are not directly involved in the patient handover process were excluded.

#### Perception Study

In the perception study, eligible medical officers were enrolled as participants upon consent and meeting the inclusion-exclusion criteria. The participants were briefed on the study, in which they were required to use the newly developed ECH tool (Figure 3) for 1 month (test period) during their work shift. Upon completing their one-month testing period, they were asked to complete the provided questionnaire (Table 1). The questionnaire consists of 10 items assessing their perceptions of using the new ECH tool. The participants were to rate each question on a 3-point Likert scale. The participants were also required to answer “Yes” or “No” to the question: “Do you like to use the ECH tool for handovers?”

### Statistical Analysis

For phase one FDM, the statistical analysis was based on the FDM method, seeking values of *d*, the percentage of consensus, and Fuzzy *A* value. In phase two, the 3-point Likert scale questionnaire uses the following scale: 0 = Disagree, 1 = Agree, and 2 = Strongly Agree. The analysis involved summing these scores to calculate the population mean (μ) and the overall percentage of agreement. These values were then compared against a Z-score percentile threshold derived from the pilot study data. The study utilised a Z-score percentile approach to establish a specific “cut-off” point for face validity. The benchmark for a “Good” outcome was explicitly defined as achieving an overall percentage of agreement greater than the Z-score percentile derived from the pilot study. Therefore, reporting the percentage is essential to demonstrate that the tool statistically exceeded this threshold and met the pre-determined acceptance criteria.

## Results

### Phase One Results

#### The Number of Expert Panels

Ten EPs that met the inclusion-exclusion criteria were enrolled in the study, forming the expert panel. They consisted of five males and five females. Most were in the 40-to-50-year age range (*n* = 7, 70%), whilst the others were in the 50-to-60-year age range (*n* = 3; 30%). The number of years of experience as an EP varied, with most having 11 to 15 years (*n* = 5; 50%), some having 6 to 10 years (*n* = 3; 30%), and the least having two to five years (*n* = 2; 20%).

#### Determining the Items of the ECH Tool Using FDM

Using a deductive approach, 20 items were identified. After the FDM, only 19 items were accepted, as summarised in Table 2. One item was rejected: “patient financial eligibility and insurance status.” When the 19 items were mapped to the ISBAR domain, the results were as follows: i) five items in Identification/Information; ii) two in Situation; iii) two in Background; iv) seven in Assessment/Action; and v) three in Recommendation. The five items in the domain of Identification/Information were “patient identification (name, age, and gender),” “length of stay in ED,” “code or end of life directive status,” “relevant past medical/surgical history,” and “allergies history.” The two items in the Situation domain were “current/working diagnosis” and “current issue.” The two items that belong to the Background domain were “presenting complaints” and “history of presenting complaints.” As for the seven items that were included in the domain of Assessment/Action, they were “vital signs,” “relevant physical examination,” “relevant bedside examination,” “relevant results of investigations,” “treatments given/procedure done,” “consulting teams,” and “changes in conditions in ED.” Finally, the three items of the Recommendation domains were “identify parts of the plan that need to be completed,” “likely disposition plan,” and “identify conflicts with consulting.”

#### The Final Design of the ECH Tool

With 19 items deemed accepted after the FDM reached consensus amongst the expert panel, the ECH tool was designed to incorporate all items. The final product of the newly developed ECH tool is presented in Figure 3.

### Phase Two Results

#### Perception Study

Referring to the perception study, the mean percentage score is 70.17%, SD ± 2.26. The distribution is left-skewed. Four statements demonstrated good agreement among participants. They are S2, S3, S7, and S9, which received scores of more than 70.17% (Table 3). The statement least agreeable was S5, which received only 65%.

## Discussion

This study aimed to develop an ECH tool using FDM. The FDM is a simple, robust technique for attaining consensus statements. For any statement to be accepted, it has to satisfy three components: i) a threshold *d*-value of less than 0.200; ii) a percentage consensus value of more than 75%; and iii) a Fuzzy *A* value of more than 0.5. In the classical Delphi method, there were several rounds of interviews. The first round requires the expert panel to suggest items to be included in the domains. The second round and subsequent rounds follow this until a consensus is achieved on the agreed item. This iterative process can take a very long time to complete.

Using the FDM, 20 essential items were identified for inclusion in the ISBAR framework. In the present study, fuzzy numbers with a 7-point Likert scale were used as the linguistic criteria. A 7-point Likert scale, rather than a 5-point Likert scale, was chosen to give the panel of experts more distinct choices and a higher level of consensus. It improves granularity and is more sensitive to changes in opinions, leading to a more accurate consensus on ambiguity.

According to the FDM, the domain Introduction contained five items: “patient identification (name, age, and gender),” “length of stay in ED,” “code or end of life directive status,” “relevant past medical/surgical history,” and “allergies history.” Initially, this domain contained six items. Nonetheless, one of the items, “patient financial eligibility and insurance status,” was rejected by the panel, as it met the threshold *d*-value of 0.279, a percentage of agreement of 50%, and the Fuzzy *A* value of 0.257.

The panel feels that the item “patient financial eligibility and insurance status” is not crucial in a government hospital, as patients do not require a high level of financial backing to receive emergency treatment. Most government hospitals in Malaysia require patients to pay only a minimal amount before treatment. Usually less than RM10. However, this financial factor might be crucial in a private medical facility. The need for insurance was also not considered a strong factor in the handover of emergency cases, as the government hospital’s policy ensures that any emergency patient receives treatment regardless of insurance status. Compared with the United States (US), having medical insurance is a very important factor in receiving treatment, even in an emergency (14).

In the Situation domain, two items that were considered to be very important were “current/working diagnosis” and “current issue” (Table 2). Both items score exceptionally well in the FDM, receiving almost identical marks in *d*-value, consensus percentage, and Fuzzy *A* value. This is quite expected, as having a working diagnosis and understanding the current issue will make the ongoing treatment go more smoothly for the receiving team. In other words, they do not need to start evaluating the patient from the beginning, which can negatively impact patient care in terms of time and energy (15).

For the domain Background, the items were “presenting complaints” and “history of presenting complaints” (Table 2); both items were highlighted as important information in the ECH. However, upon further analysis, “presenting complaints” was noted to receive a better score in the *d*-value and Fuzzy *A* value, indicating its importance over “history of presenting complaints.” This is an interesting finding, and it is observed that acknowledging the chief complaint will help the treating doctor who receives the handover focus more promptly on the patient’s problem.

Among the seven items in the Assessment domain, the item on “changes in conditions in ED” receives the highest agreement score from the expert panel. It received a perfect score in *d*-value, with 100% consensus and a Fuzzy *A* value of 0.967. Recognising the patient’s response after treatment is an important indicator that guides further management. It is also interesting to note that “vital signs” does not receive the perfect score as “changes in conditions in ED,” even though doctors were taught that vital signs are the most vital in any patient. According to Brekke and colleagues (16), monitoring of vital sign trend changes is very important in emergency patient care. This is an interesting topic that needs further research and evaluation.

Of the three items in the Recommendation domain, “identify parts of the plan that need to be completed” is most highly agreed upon by the panel, followed by “likely disposition plan” and “identify conflicts with consulting.” This is quite understandable to include in the ECH, as knowing the plans for completion will make the job of the receiving team more organised, concise, and relevant, with readily accessible clinical recommendations for further care in an ED (17). It also saves time, especially in a busy ED.

In comparison, the available ISBAR is a general mnemonic/framework. In contrast, this present study’s FDM output is a validated, item-specific tool tailored for the Malaysian ED context. Key differences include:

- Contextual relevance: Unlike generic models, this tool excludes “patient financial eligibility/insurance status,” which the experts rejected (threshold *d* = 0.279) as irrelevant for emergency care in Malaysian government hospitals, contrasting with US-based models where this is often standard; and
- Granularity: The tool standardises specific critical data points often missed in generic ISBAR handovers, such as “changes in condition in ED” (which received 100% expert consensus) and “identify parts of the plan that need to be completed.” This ensures the receiving team knows exactly what happened during the shift and what remains pending, rather than just the patient’s status.

Regarding the ED medical officer’s perception of the new ECH tool, the mean score was 70.17% (SD ± 2.26), with all agreement. It indicates that they observed the tool to be an effective, practical instrument suitable for the fast-paced ED environment. While a 70.1% agreement rate might appear moderate initially, this is interpreted as a positive and statistically significant outcome for the following reasons:

- Statistical validation: The 70.1% agreement exceeded the calculated *Z*-score percentile benchmark of 61.2%, which was established using data from the pilot study. According to the study’s pre-determined validation criteria, this classifies the tool as “Good.”
- Central tendency bias: A “central tendency bias” was observed where participants selected the neutral middle option (score of 1 on a 0–2 scale) rather than “Strongly Agree.” This often reflects a hesitation to appear extreme rather than outright rejection.
- Change management: The resistance (approximately 30%) likely stems from the shift from verbal/memory-based handovers to a written/structured tool, which participants initially perceived as “more work” or “too comprehensive,” as noted in open feedback. This highlights the need for continued training and perhaps digitisation to ease the documentation burden, rather than a failure of the tool’s content.

Four statements stand out about the ECH tool: easy to use, appropriate, beneficial, and improving the handover process are the top features. These results confirm that the ECH tool meets clinical relevance and operational feasibility. However, the least agreeable statement was that the ECH tool is more comprehensive than the previously practised handover. This could be due to the items in the new ECH tool being almost identical to the usual handover practice. Nevertheless, having excessive items in the ECH tool might make it more complicated and less compliant. In fact, some wrote open comments on the questionnaire, such as “When many things to fill, it will be difficult to ensure compliance.” Therefore, it is considered appropriate to have a balanced ECH tool for ensuring compliance and effectiveness.

The ECH tool improves clinical efficiency by addressing the root causes of handover delays and errors. This is achieved through:

- Reducing repetition: By explicitly documenting “current issues” and “changes in condition in ED,” the receiving team does not need to re-evaluate the patient from scratch, saving time and cognitive energy;
- Streamlining workflow: The inclusion of the item “identify parts of the plan that need to be completed” allows the incoming team to immediately prioritise outstanding tasks (e.g., pending investigations or referrals), making the transition more organised and actionable; and
- Standardisation: By standardising the 20 critical items, the tool reduces variability and the risk of information omission, which is a known cause of delayed diagnosis and unnecessary repetition of investigations.

### Limitations

This study was conducted in a single centre, with the expert panel consisting of EPs from the same hospital. The determination of the items in the ECH tool is based on the ED climate of that particular centre. Note that this may not be the case at other hospitals in the region. Consequently, this may limit generalisability to other EDs with different patient populations, resources, or operational models. Whilst the outcome of this study provides a useful tool for that institution, it may not be a perfect fit for other hospitals.

Secondly, as a single-centre study, the respondents in the perception study may also have been biased since they were exposed to the same case mix. This might reduce the generalisability of the ECH tool. Thus, expanding this study amongst ED medical officers from other institutions will enhance the generalisability of the tool.

Thirdly, the perception study uses a 3-point Likert scale, which may bias results toward central tendencies. This was observed in 8 of 10 statements, with the majority of respondents providing a midpoint score. Hence, to avoid this, an even-number Likert scale, such as a 2-point or 4-point scale, may be used.

## Conclusion

The development of the ECH tool marks a significant advancement in improving communication practices. By integrating the ISBAR framework and refining its components through the FDM, the tool successfully identifies and standardises 19 critical items necessary for safe and effective patient handovers. Furthermore, its structured format, following the ISBAR framework, addresses the long-standing issue of inconsistent and unstandardised handovers that can compromise patient safety. Remarkably, the ECH tool enhances clarity and continuity of care and fosters a culture of accountability and standardisation among healthcare providers. While further research is required to assess its broader applicability and long-term impact on clinical outcomes, the tool represents a promising step toward elevating the quality and reliability of emergency care communication across Malaysian EDs.

## Figures and Tables

**Figure 1:**
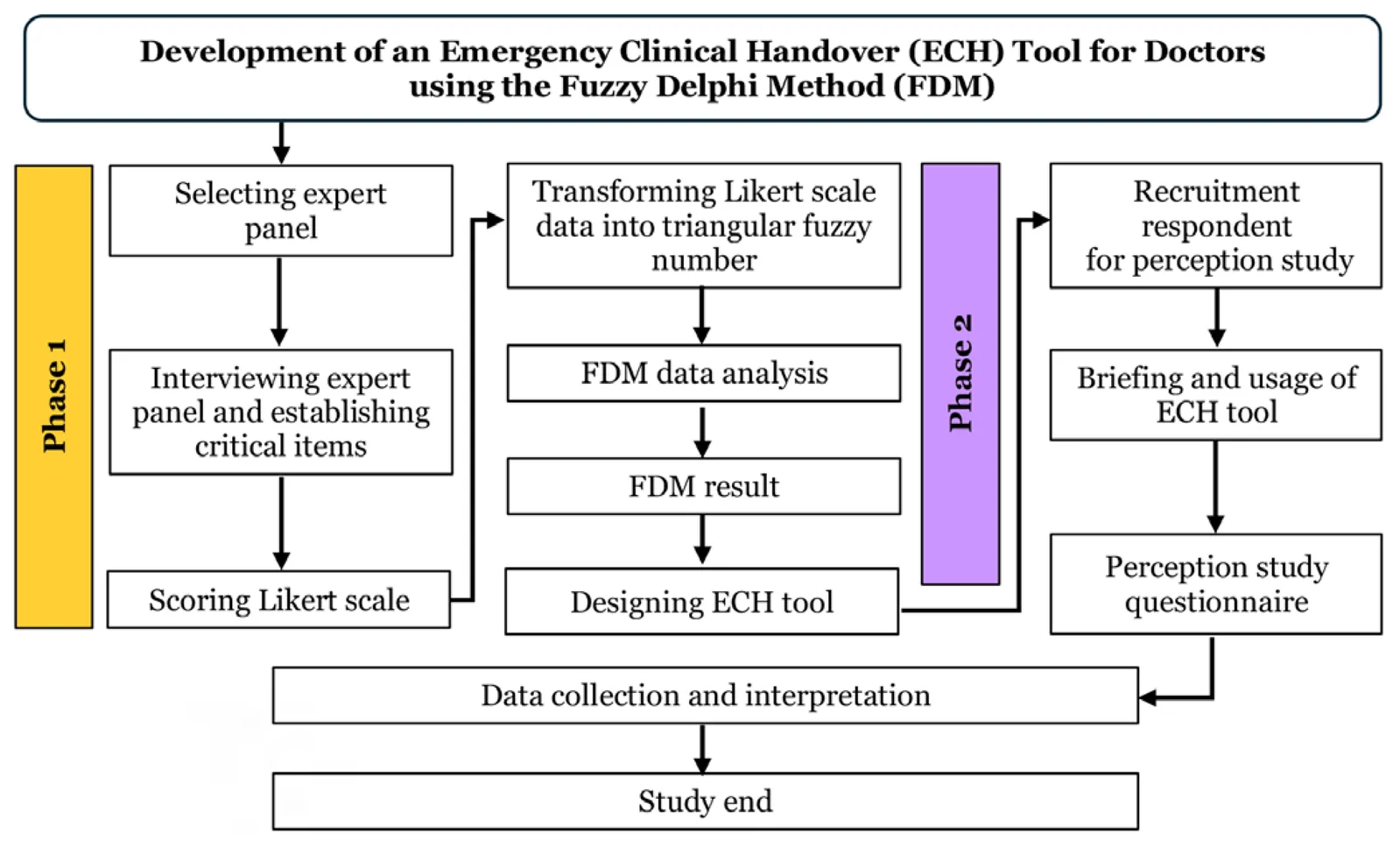
Study flow of the development of an ECH tool using FDM

**Figure 2:**
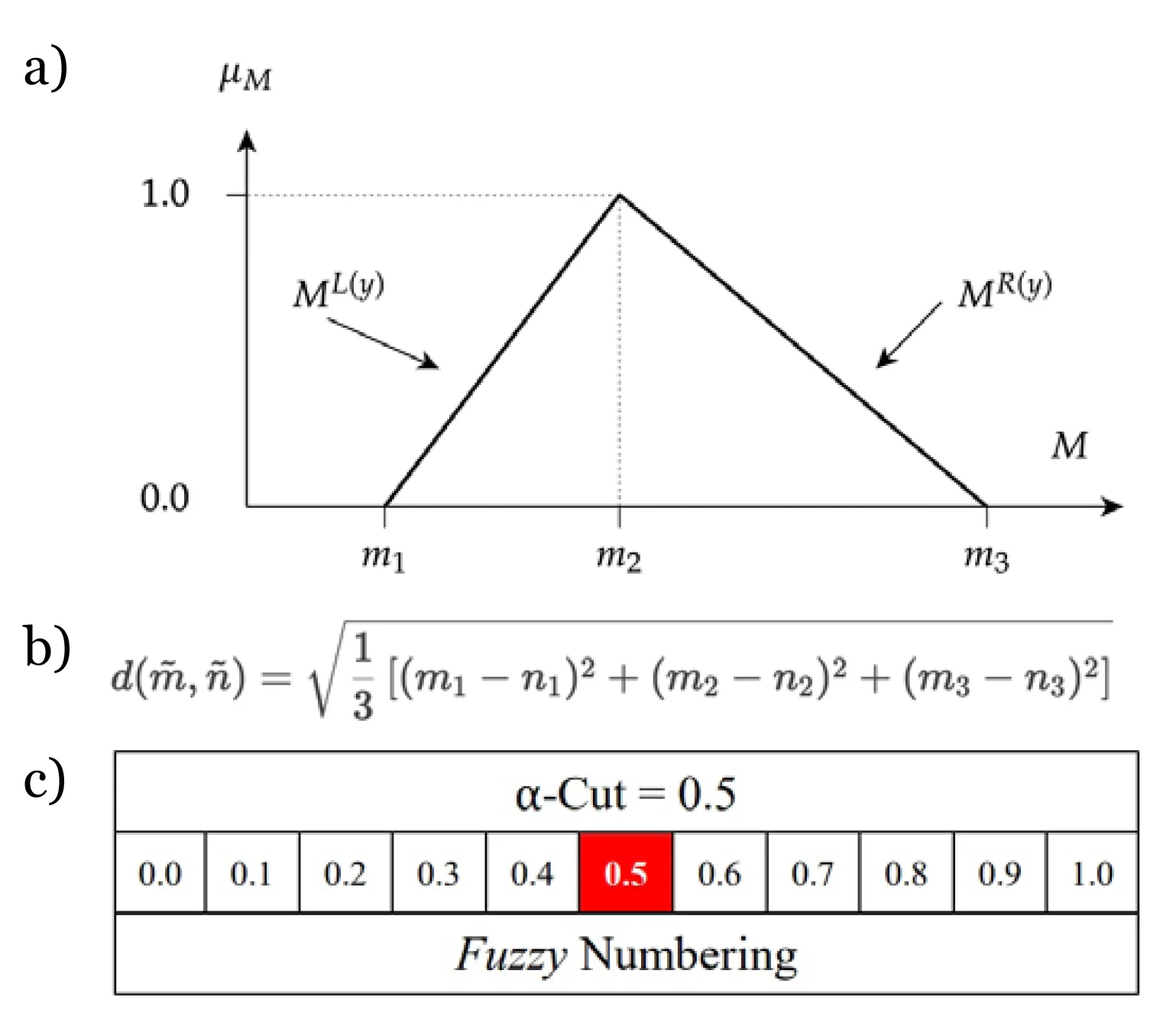
Formula used in FDM: a) triangular fuzzy numbers; b) formula for *d*-value; and c) formula for Fuzzy A value (11, 13)

**Figure 3:**
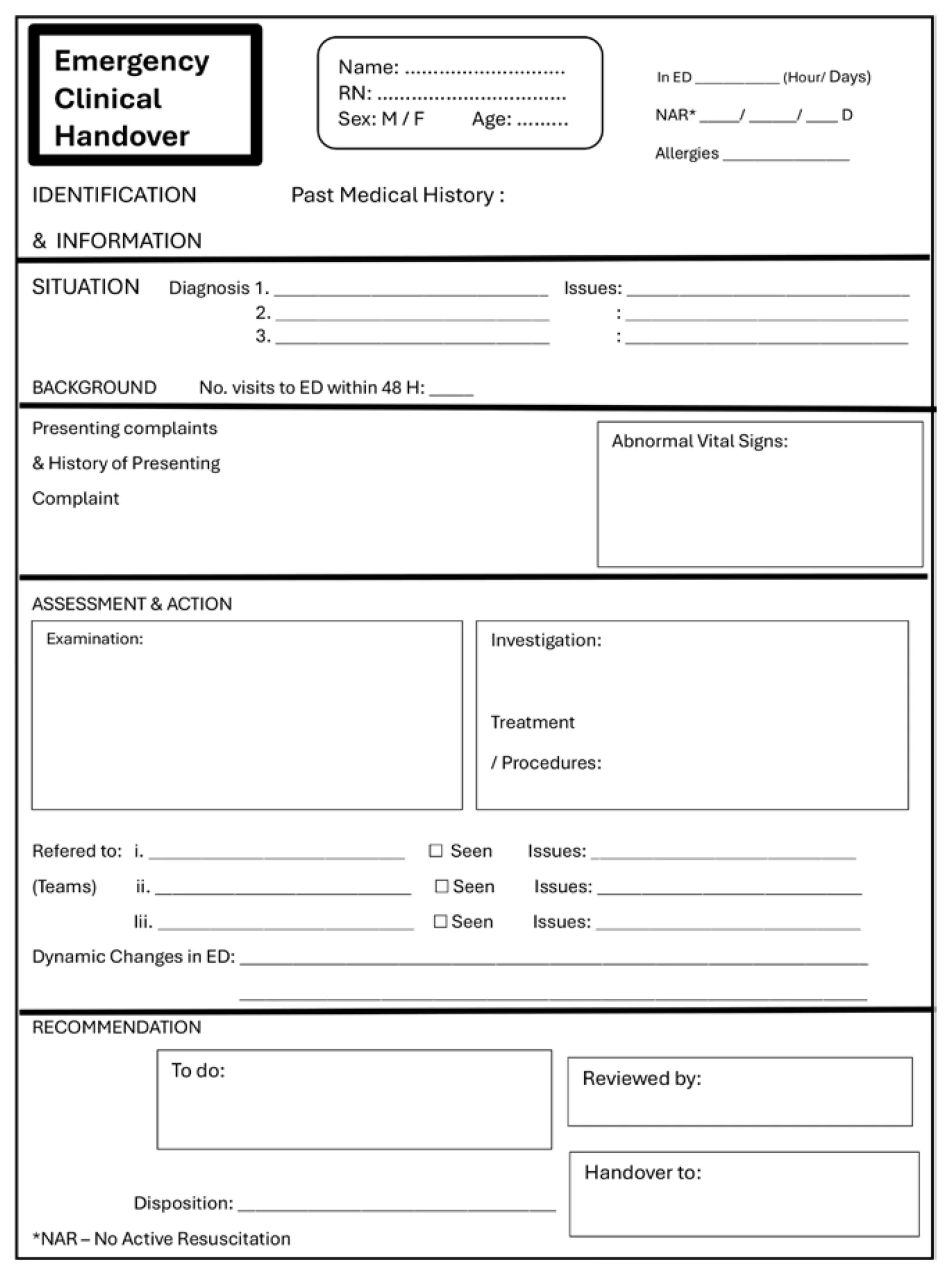
The newly developed ECH tool

**Table 1:** Assessment of perception amongst ED medical officers towards the ECH tool

No.	Statement	Likert score			Total marks (%)
		0	1	2	
		Frequency (*n*)			
S1	The instruction to use the ECH tool is adequate.	0	20	10	40/60 (66.6)
S2	Each element in the ECH tool is easy to understand.	0	15	15	45/60 (75.0)
S3	The elements in the ECH tool are appropriate.	0	14	16	46/60 (76.6)
S4	The ECH tool is easy to use and user-friendly.	0	18	12	42/60 (70.0)
S5	The ECH tool is more comprehensive than previously practised handover.	1	19	10	39/60 (65.0)
S6	The ECH tool is relevant in the ED setting.	0	18	12	42/60 (70.0)
S7	The ECH tool is beneficial in the ED setting.	0	16	14	44/60 (73.3)
S8	The ECH tool is feasible in the usual timeframe of a handover in ED.	0	20	10	40/60 (66.6)
S9	The ECH tool improves the handover process in ED.	0	17	13	43/60 (71.6)
S10	The ECH tool is favoured to be used again for handovers.	0	20	10	40/60 (66.6)
			**Yes**	**No**	
I like to use the ECH tool for handovers			30	0	

**Table 2:** Item-level Fuzzy Delphi outcomes

Item	*d* (threshold)	Agreement (%)	*A* (defuzzified)	Decision
**Identification**				

Patient identification (name, age, and gender)	0.027	100	0.957	Accept
Length of stay in ED	0.103	90	0.910	Accept
Patient financial eligibility and insurance status	0.279	50	0.257	Reject
Code or end of life directive status	0.166	90	0.863	Accept
Relevant past medical/surgical history	0.073	100	0.927	Accept
Allergies history	0.103	90	0.910	Accept

**Situation**				

Current/working diagnosis	0.027	100	0.957	Accept
Current issue	0.027	100	0.957	Accept

**Background**				

Presenting complaints	0.027	100	0.957	Accept
History of presenting complaints	0.064	100	0.937	Accept

**Assessment**				

Vital signs	0.087	90	0.930	Accept
Relevant physical examination	0.027	100	0.957	Accept
Relevant bedside examination	0.049	100	0.947	Accept
Relevant results of investigations	0.087	90	0.930	Accept
Treatment given/procedure done	0.027	100	0.957	Accept
Consulting team	0.103	90	0.910	Accept
Changes in conditions in ED	0.000	100	0.967	Accept

**Recommendation**				

Identify parts of the plan that need to be completed	0.064	100	0.957	Accept
Likely disposition plan	0.181	90	0.860	Accept
Identify conflicts with consulting	0.181	90	0.860	Accept

**Table 3:** Face validation response with overall percentages, μ2, overall total, total per panellist and total per question calculated

Panellist	Years of ED experience	S1	S2	S3	S4	S5	S6	S7	S8	S9	S10	Total per panel	I like to use the ECH Tool
1	7	2	2	2	1	1	1	2	1	2	2	16	Yes
2	4	1	1	1	1	1	1	1	1	1	1	10	Yes
3	8	2	2	2	2	2	2	2	2	2	2	20	Yes
4	5	1	1	1	1	2	1	1	1	1	1	11	Yes
5	6	2	2	2	2	1	2	2	1	2	1	17	Yes
6	7	1	1	1	1	1	1	1	1	1	1	10	Yes
7	4	1	1	1	1	0	1	1	1	1	1	9	Yes
8	10	1	1	1	1	1	1	1	1	1	1	10	Yes
9	7	1	1	1	1	1	1	1	1	1	1	10	Yes
10	7	1	2	2	2	2	1	1	1	2	2	16	Yes
11	7	1	1	2	2	1	1	1	2	1	1	13	Yes
12	7	2	2	1	1	1	1	1	1	1	1	12	Yes
13	6	2	1	1	1	1	2	2	1	1	1	13	Yes
14	5	2	2	2	2	2	2	2	2	2	2	20	Yes
15	8	1	2	1	1	1	2	1	1	1	1	12	Yes
16	6	2	2	2	2	1	2	2	2	2	2	19	Yes
17	9	1	1	2	1	1	2	2	2	2	1	15	Yes
18	8	1	1	1	1	1	1	1	1	1	1	10	Yes
19	7	1	1	2	2	1	1	1	1	1	1	12	Yes
20	7	1	2	2	1	2	1	1	1	1	1	13	Yes
21	9	1	1	1	1	1	1	2	2	2	1	13	Yes
22	7	1	1	1	1	1	1	1	1	1	1	10	Yes
23	5	2	2	2	1	2	2	2	2	2	2	19	Yes
24	9	2	2	2	2	2	2	2	2	2	2	20	Yes
25	9	1	1	1	1	1	1	1	1	1	1	10	Yes
26	7	1	1	2	2	1	1	2	1	2	2	15	Yes
27	9	1	2	1	1	1	1	1	1	1	1	11	Yes
28	5	1	2	2	2	2	2	2	1	1	1	16	Yes
29	6	1	2	2	2	2	2	2	2	2	2	19	Yes
30	5	2	2	2	2	2	2	2	2	2	2	20	Yes
Total per question		40	45	46	42	39	42	44	40	43	40	Overall total 421/600	Overall percentage 70.1%
Percentage agreement per question (%)		66	75	76	70	65	70	73	66	71	66	μ_2_ = 14.0	μ: Population mean
